# Transmembrane Protein-184A Interacts with Syndecan-4 and Rab GTPases and Is Required to Maintain VE-Cadherin Levels

**DOI:** 10.3390/cells14110833

**Published:** 2025-06-03

**Authors:** Leanna M. Altenburg, Stephanie H. Wang, Grace O. Ciabattoni, Amelia Kennedy, Rachel L. O’Toole, Sara L. N. Farwell, M. Kathryn Iovine, Linda J. Lowe-Krentz

**Affiliations:** 1 Department of Biological Sciences, Lehigh University, Bethlehem, PA 18015, USA; lmt318@lehigh.edu (L.M.A.); grace.ciabattoni@gmail.com (G.O.C.); ameliakennedy2003@gmail.com (A.K.); rachellaniotoole@gmail.com (R.L.O.); slnfarwell@gmail.com (S.L.N.F.); mki3@lehigh.edu (M.K.I.); 2 Department of Microbiology, New York University Grossman School of Medicine, New York, NY 10016, USA; 3 Galveston National Laboratory, University of Texas Medical Branch, Department of Microbiology and Immunology, University of Texas Medical Branch, Galveston, TX 77555, USA; 4 Independent Researcher, Pittsburgh, PA 15201, USA

**Keywords:** VE-cadherin, TMEM184A, syndecan-4, endothelial cells, angiogenesis

## Abstract

VE-cadherin (VE-cad) membrane stability and localization regulates adhesion formation and actin cytoskeleton dynamics in angiogenesis and vascular remodeling and requires the heparan sulfate proteoglycan (HSPG), Syndecan-4 (Sdc4). This study characterizes the interactions of the heparin receptor, Transmembrane protein-184A (TMEM184A), and Sdc4 in bovine aortic endothelial cells (BAOECs) and the regenerating Zebrafish (ZF) caudal fin and measures the effect of siRNA TMEM184A KD (siTMEM) and TMEM184A overexpression (TMEM OE) on VE-cad levels and localization in confluent and sub-confluent cultured BAOECs. Additionally, we examined the effect of siTMEM on key Rab GTPase trafficking regulators and migrating BAOECs in scratch wound healing assays. We demonstrated that TMEM184A and Sdc4 colocalize in BAOECs and that Sdc4 OE increases colocalization in an HS chain dependent manner, while both Tmem184a and Sdc4 cooperate synergistically in ZF fin angiogenic and tissue repair. We also showed that siTMEM decreases VE-cad membrane and cytoplasmic levels, while increasing scratch wound migration rates. However, TMEM OE cells show increased vesicle formation and VE-cad trafficking and membrane recovery. These findings characterize TMEM184A-Sdc4 cooperation in angiogenesis and indicate a dual function of TMEM184A in signaling and trafficking in vascular cells that promotes VE-cad recovery and membrane localization.

## 1. Introduction

In vascular research with cultured cells, it has long been recognized that exogenous heparin modifies both endothelial and smooth muscle cell proliferation and the endothelial inflammatory stress response [1,2,3,4,5,6,7]. Endothelial cell (EC) remodeling and controlled proliferation in inflammation are essential to vascular wound healing and repair in a developed organism [8,9] and reparative angiogenesis can improve recovery from surgical treatments of cardiovascular disease [10]. There is clear evidence that Transmembrane Protein-184A (TMEM184A), a heparin receptor, is required for the heparin-induced MAPK signaling changes observed in cultured cells. Signaling through TMEM184A dampens cell proliferation and reduces stress fiber formation in the presence of growth factors or inflammatory mediators [1,4].

Repair of damaged endothelium and reparative angiogenesis typically require Vascular Endothelial Growth Factor (VEGF) which results in EC proliferation. It also results in increased survival of cells, increased migration, and modulation of endothelial cell–cell adhesion [11,12,13]. Angiogenesis involves VEGF activation of VEGFR2 and in mice requires the heparan sulfate proteoglycan (HSPG) Syndecan-2 (Sdc2), an HSPG also required for zebrafish (ZF) vascular development [14,15]. Many studies of EC migration indicate the involvement of Syndecan-4 (Sdc4) [16,17], an HSPG recently shown to be involved in directed cell migration through specialized Integrin recycling [18,19,20] and required for VE-cadherin (VE-cad) internalization in murine wound healing and VEGF mediated pathological angiogenesis [21,22].

In the ZF developmental angiogenesis system, VE-cad plays a role in outgrowth of new vessels and facilitates anastomosis of separate growing vessels to form complete vascular networks [23,24]. Previous studies of VE-cad in vascular cells have shown that tight regulation of VE-cad trafficking to and from adherens junctions (AJs) is required for vessel sprouting in angiogenesis as cells decouple, proliferate, and migrate toward growth signaling factors [11,21,24,25], and VE-cad internalized through clathrin mediated endocytosis leads to VE-cad C-terminal domain proteolytic cleavage and protein degradation [26]. In turn, rapid recycling of intact VE-cad in angiogenesis has been shown to depend classically on Rab4 and Rab11a GTPase redelivery of VE-cad to the membrane surface [21,27]. In early investigations of TMEM184A (also referred to as Sdmg1 in exocrine tissues), observations in IHC and IF showed discreet puncta of TMEM184A in pancreatic, salivary, and mammary tissues and increased apical cytoplasmic puncta in mammary tissue excised from pregnant and lactating mice [28]. It follows that TMEM184A, the vascular version of Sdmg1 and novel heparin receptor, may maintain a dual function in trafficking key proteins, including VE-cad in addition to initiating heparin-induced signaling. Consistent with that idea, Tmem184a KD in ZF embryos showed a decrease in total VE-cad in proliferating stalk cells [29].

Characterization of Tmem184a (protein notations appear lower case, nucleic acids italicized in accordance with ZF nomenclature) in vivo in the adult regenerating ZF caudal fin showed increased vascular EC proliferation with Tmem184a morpholino (MO)-mediated gene knockdown (KD) injections concurrent with reduced vascular and tissue outgrowth, while cell proliferation decreased with heparin injection of Tmem184a KD fins [30]. In a study of angiogenesis in the developing ZF embryo, Tmem184a KD resulted in greater endothelial cell proliferation and abrogation of intersegmental vessel (ISV) completion. In addition, removal of the putative heparin-binding domain also reduced ISV completion [29]. These results separate the proliferation of angiogenesis from vascular outgrowth, that is angiogenesis, suggesting that endogenous TMEM184A signaling and protein interactions in wild-type vascular cells limit proliferation to promote cell organization in vessel formation.

These results led us to ask whether ZF Tmem184a may modulate angiogenesis through Sdc4 interactions and whether changes in TMEM184A expression in bovine aortic ECs (BAOECs) affect VE- cad levels through modulation of trafficking and actin cytoskeleton dynamics in collective cell migration. In this report, we identified Sdc4—TMEM184A interactions in BAOECs and observed that synergy of subthreshold *tmem184a* and *sdc4* MOs contributed to a ZF vascular phenotype. In ECs, we have shown that TMEM184A expression affects relative levels of VE-cad at the cell surface and in vesicles, and that TMEM184A colocalizes with Rab4 and Rab11a GTPases. Collectively, these data support that TMEM184A and Sdc4 interactions regulate cell adhesion and coordinate cell movement in EC remodeling and angiogenic repair.

## 2. Materials and Methods

### 2.1. Zebrafish Strains, Housing, and Husbandry

The animal model that was used for this study is the ZF (Danio rerio) C32 strain [31]. The *TG*(*fli1: eGFP^y1^*) transgenic line from Lawson and Weinstein [32], was utilized for visualization of caudal fin regeneration. Male and female siblings were included in cohorts for this study. All animal experiments were conducted in accordance with the recommendations in the Guide for the Care and Use of Laboratory Animals from the National Institutes of Health. This protocol was approved by Lehigh’s Institutional Animal Care and Use Committee (IACUC) (Protocol # 172, initially approved 17 November 2014 and most recently renewed as protocol 172 approved 19 July 2024). Our on-site study location, Lehigh University’s Animal Welfare Assurance Number is A-3877-01. All experiments were conducted with the implementation of Tricaine anesthesia to minimize animal pain and discomfort.

ZF were housed in a Pentair re-circulating water system in 3 L and 10 L tanks containing 12–15 and 20–30 fish per tank, respectively. Tanks were maintained on a 14:10 light: dark cycle and the room temperature was kept at 27–29 °C. A 10% water exchange was performed with daily water quality monitoring. Nitrogen levels were optimized by a biofilter and dosed automatically to maintain conductivity (400–600 μs) and pH (6.95–7.30). Sequential filtering of re-circulating water was achieved through the use of pad filters, bag filters, and a carbon canister. Water was passed through ultraviolet light for sterilization. Fish diets were maintained with one feeding of fresh brine shrimp (INVE artemia cysts) and one daily feeding of flake (Zebrafish Select Diet, Aquaneering Inc., San Marcos, CA, USA).

### 2.2. Morpholino Injections

ZF fins were amputated (50% of the caudal fin length). At 72 h post amputation (hpa), the identified amount of MO was injected into the dorsal half of the regenerating fin as described [33]. MO microinjections (*sdc4* ATG translational start site blocker sequence (5′-TGAGGTAAACTTTCAACATCTTCTC-3′), *tmem184a* ATG translational start site blocker 166 (5′-CTGAGAGTAGTTTCATTCATCCTGA-3′), and control MO (5′-CCTCTTACCTCAGTTACAATTTATA-3′) all labeled with Lissamine rhodamine) (Gene Tools) were administered to the tip of each fin ray from the dorsum of the fin to the midline 3 days post amputation (dpa), and both injected and uninjected fin rays were electroporated (Nepal Gene CUY21EDIT) to encourage MO diffusion across cell membranes (as previously described [30]). Fins were harvested at 4 dpa into 4% PFA and fixed overnight at 4 °C. Fixed fins were mounted in 50% glycerol for confocal or compound microscopy.

### 2.3. Fin Imaging Measurements and Statistical Analysis

Fins were imaged with a Zeiss LSM 800 confocal microscope (Carl Zeiss Microscopy, LLC, White Plains, NY, USA), 1024 × 1024 acquisition, 5× objective at a central z-position to fin thickness to maximize the view of the vasculature. The Nikon Eclipse 8oi compound microscope (Nikon, Minato City, Tokyo, Japan) with a GFP filter and NIS Elements software (BR 4.60.00) was used to obtain fin images of the 0.25 mM subthreshold synergy experiments at a 5× objective. Fin images of injected and uninjected sides from each fish within a representative group were aligned to measure total vessel and fin outgrowth from the amputation plane to the tip of the vessels and tissue outline. Measurements were taken in μm of the third and fourth rays of the injected and uninjected sides. The percentage difference for outgrowth of vessel and tissue was calculated as a ratio of injected μm/uninjected μm measurements [30]. Statistical significance between the control and MO injected groups was determined with a one tailed, student t-test, for a heteroscedastic sample.

### 2.4. Materials—Antibodies

Antibodies used in the study are listed in Table 1.

### 2.5. Culturing and Transfections

Two separate primary cell lines of BAOECs (Cell Applications, San Diego, CA, USA), passaged 6–10 times, were used for this study. Cells were passaged 1:2 to continue cultures in 1X PBSE, 0.1 mM EDTA (Fisher, Scientific, Pittsburg, PA, USA) and 1X Trypsin/EDTA solution (Cell Applications) with Minimum Eagles Medium (MEM) (Sigma-Aldrich, St. Louis, MO, USA), 10% HI-FBS (Gibco, now part of Thermo Fisher Scientific, Waltham, MA, USA), 4 mM L-Glutamine (Cell Applications), 1% Penicillin-Streptomycin (Sigma-Aldrich), 1% MEM Amino Acids (Sigma-Aldrich), 1% Sodium Pyruvate (Sigma-Aldrich). Cells were seeded in 100 mm and 150 mm dishes coated with 0.2% Porcine Gelatin (Sigma-Aldrich) and kept in a humidity incubator covered with 10–12 mL (100 mm) and 20–25 mL (150 mm) of MEM at 37 °C, 5% CO_2_. BAOEC lentiviral cell lines were established employing lentiviral constructs (VSV-G and GAG POL) with Sdc4 murine constructs (wildtype Syndecan-4-HA (Sdc4-HA) and Syndecan-4-HA-ΔGAG (Sdc4-HA-ΔGAG) lacking HS chains) generously donated by James Whiteford [21]. Constructs were cultured with selection and purified using the ZymoPURE II maxiprep kit (Zymo Research Corporation, Irvine, CA, USA, catalog # D4202), then packaged in cultured HEK293T cells (ATTC, Manassas. VA, USA) with OptiMEM (Cell Applications) and 0.3 μM polyethyleneimine (Sigma-Aldrich) in standard culturing conditions with 10% CO_2_. Stable virus was extracted over a three-day period in MEM but without Penicillin/Streptomycin, and sterile filtered with a 0.45-micron filter. Filtered media containing lentivirus was mixed with Polybrene (Thermo Fisher, Waltham, MA, USA) at a concentration of 8 μg/mL and laid directly over 30–40% confluent passage 4 BAOECs. Cells were then cultured as previously described.

Prior to electroporation and seeding for end-point harvests, cells were trypsinized and counted with a hemocytometer. Cells were seeded to achieve desired confluence endpoints in harvests, 60–80% confluence or >90% confluence for comparison in assays. BAOECs were trypsinized, counted and pelleted in an IEC Clinical centrifuge at 300× *g*, then washed in sterile 1X PBS and resuspended in Hepes Buffered Saline (HeBS) at a concentration of 5 × 10^6^ cells per ml for electroporation. Cells were mixed gently with control siRNA or Bovine TMEM184A siRNA (Santa Cruz Biotechnology, Dallas, TX, USA, 3.4 ng/μL) or TMEM184A-tGFP plasmid DNA (OriGene, Rockville, MD, 10 ng/μL) for each 200–400 μL reaction and electroporated in chilled 0.2 mm gap electroporation cuvettes using the BioRad (Hercules, CA, USA) Gene Pulser Square Wave preset protocol settings for mammalian, CHO cells with one pulse (Electroporation settings adapted from [4]). Cells were incubated on ice for 5–10 min prior to reseeding, fed the next day, then harvested within 48 h post electroporation (hpe). A mock control electroporation was used to optimize electroporation parameters for cell survival and as a control measure for TMEM184A-tGFP electroporations as recommended [4].

### 2.6. Immunofluorescent Staining

Cells were fixed in 4% Paraformaldehyde (PFA) (ThermoFisher) to porcine gelatin coated glass coverslips for 20 min at room temperature (RT) with constant rocking. Cells were washed in 2 turns of 1X PBS for 5 min and permeabilized in 0.1% Triton X-100 1X PBS—0.3% Triton X-100 1X PBS blocking and permeabilization buffer, 5% normal donkey serum (NDS) (Sigma-Aldrich), 1% bovine serum albumin (BSA) (Sigma-Aldrich) for 10–30 min. In assays utilizing 10 min permeabilization intervals to preserve visualization of the membrane, cells were blocked for an additional 20 min in buffer without detergent. Slips were washed in 1 turn of 1X PBS and incubated overnight at 4 °C in primary antibody solutions. On day two, slips were washed in 3 turns of 1X PBS for 5 min at RT with constant rocking, incubated in secondary antibody solutions for 1 h at 37 °C, and rinsed in three turns of 1X PBS prior to mounting. Slips were imaged with the Zeiss Confocal LSM 800 (Carl Zeiss Microscopy, LLC, White Plains, NY, USA) using a 1024× 1024 acquisition, 63× objective in a series of 10–12 z-stacks. Images show cells incubated as below (in Table 2) and mounted in Vectashield (Vector laboratories, Inc., Newark, CA, USA). or mounted in Vectashield with DAPI mounting media.

### 2.7. Co-Immunoprecipitation and Western Blotting

Gels were poured and polymerized at 12%, 10%, and 8% acrylamide for running immunoprecipitation (IP) samples, whole cell lysate (WCL), and subcellular fractions on SDS-PAGE, respectively. Gels were run using the Hoeffer mini gel system at 80 V, 30 mA for 120–150 min and transferred to a nitrocellulose membrane using a semi-dry transfer system (Hoeffer) with 55 V, 150 mA for 15–40 min. Membranes were washed for 15 min in 1X Tris buffered Saline pH 7.5 and blocked for one hour at RT—overnight at 4 °C in Hi-Blotto (1X Tris Buffered Saline pH 7.5, 5% non-fat dry milk, 5% normal donkey serum, 0.1% Tween-20, 0.1% NaN_3_). Blots were incubated in primary antibody solutions in 1X TBST (1X Tris Buffered Saline pH 7.5, 0.1% Tween-20) at the following dilutions overnight at 4 °C with constant rocking. Blots were washed in 3 turns of 1X TBST for 5 min and incubated in 1X TBST secondary antibody solutions at RT with constant rocking and imaged with the BioRad Fluorescence ChemiDoc.

Cells grown on 150 mm dishes were harvested at 70% confluency into 2% CHAPS/1X PBSE, 5 mM EDTA, 1X HALT (1 mL/150 mm dish), transferred to chilled microcentrifuge tubes, and incubated on ice for 30 min (cell lysis). The following protocol was adapted from our previous study [3] and an online sourced CHAPS immunoprecipitation protocol (available from FIVEphoton Biochemicals, San Diego, CA, USA, 2016). Lysates were pre-cleared with Affinity protein IgG red gel beads (Sigma-Aldrich, 50 μL beads: 1 mL lysate), equilibrated with three cold lysis buffer washes, by rocking for one hour at 4 °C on a rotating rocker. Pre-cleared lysates were centrifuged at 9600× *g* for 10 min, 4 °C and supernatant was transferred to chilled microcentrifuge tubes. Protein concentrations of pre-cleared lysates were determined with the Pierce BCA Protein Assay kit (Thermo Fisher). Protein from a 300 μL aliquot of each sample was precipitated in a chilled microcentrifuge tube with 10% TCA for 30 min to overnight to serve as an input sample [4]. A calculated volume of Sdc4 mouse or TMEM184A rat antibody to achieve 2 μg antibody: 500 μg of total protein was added to designated immunoprecipitation (IP) lysate, keeping one tube of lysate without added antibody to serve as a bead only (BO) control. BO and IP samples were rocked for one hour at 4 °C, then centrifuged for 1 min at 9600× *g*, 4 °C and transferred to chilled and washed IgG red gel beads (50 μL: 1 mL of sample). Samples were rocked for an additional hour at 4 °C, then centrifuged for 3 min at 9600× *g*, 4 °C. Supernatant (unbound protein) was TCA precipitated (10%) on ice for 30 min—overnight. Beads were washed 3 times with 1 mL 0.002% Tween-20 1X PBSE, spinning for 3 min at 9600× *g*, 4 °C. Washed beads were boiled in two turns of 2X SDS sample buffer for 15 min at 90–95 °C to achieve a double elution of the bound protein. Input and unbound fraction protein pellets were solubilized in sample buffer and samples were run on 12% SDS-PAGE to confirm pull downs and co-immunoprecipitations with immunolabeling using the primary and secondary antibodies in Table 3.

### 2.8. WCL Harvest and Cell Fractionation

To prepare whole cell lysate, cells were harvested into 2X sample buffer directly or 2% CHAPS/PBSE with 0.5 mM EDTA and 1X HALT protease inhibitor, 5 × 10^6^ cells/mL of buffer, vortexed and incubated on ice for 30 min—overnight at −80 °C prior to standardization. To prepare lysates for subcellular fractionation (adapted from [27]), cells grown to 60–70% confluence on 100 mm dishes were scraped into 300 μL of 0.2% CHAPS lysis buffer (0.2% CHAPS PBSE 5 mM EDTA, 1% Triton X-100, 1X HALT protease inhibitor, 1 mM PMSF, pH 7.4) for control siRNA and siTMEM lysates or 0.5% Tris lysis buffer (Tris 10 mM, NaCl 140 mM, 5 mM EDTA, 0.05% SDS, 1% Triton X-100, 1X HALT protease inhibitor, 1 mM PMSF, pH 7.4) for cells electroporated in buffer only or TMEM-tGFP, incubated on ice for 20 min, and vortexed once vigorously for 5 s prior to standardization. Lysates were centrifuged at 13,000× *g* for 12 min to separate the soluble from the insoluble fraction and the supernatant was transferred to a chilled microcentrifuge tube (cytoplasm fraction). The remaining pellet was solubilized in 100 μL of 2% CHAPS PBSE with setting 8–10 vortexing (membrane fraction). Protein was precipitated from whole and subcellular CHAPS cell lysates with 10% TCA incubations on ice for 30 min—overnight at 4 °C. Samples were centrifuged at 9600× *g*, 4 °C for 10 min. Protein pellets were washed once with 1 mL of sterile water and centrifuged for 1 min at 9600× *g*. Water was aspirated, pellets were air dried and resuspended in 2X sample buffer (equilibrated with Tris HCl pH 8.8), and samples were boiled for 10–15 min at 90–95 °C for Western blotting. Samples obtained with Tris buffers were solubilized in equal parts of 2X sample buffer. Most blots were run on poured 8% gels or on poured 10% gels. The blots were incubated with antibodies shown in Table 3.

### 2.9. RNA Purification and RT-qPCR

BAOECs were transiently transfected with either TMEM184A siRNA or control siRNA following the established electroporation protocol and maintained following the established subculturing conditions for 24–30 h and harvested while cells were under 65% confluence in pairs from the same line and passage. RNA was isolated from 100 mm dishes of TMEM184a siRNA or control siRNA cells in parallel using 500 μL of Trizol. 100 μL of chloroform was added to lysates, incubated for 3 min and transferred to phase lock tubes. Samples were centrifuged at 12,000× *g* for 15 min at 4 °C. The aqueous phase was transferred to a separate tube where a DNase I recombinant digest was performed (Roche, Basel, Switzerland, 04716728001) for 30 min at 37 °C. 250 μL of isopropanol was added to the aqueous phase, mixed and incubated for 10 min at 4 °C. Samples were centrifuged for 10 min at 4 °C. The supernatant was removed and pellets washed 3 times in 75% EtOH and centrifuged for 5 min at 7500× *g* and 4 °C. Pellets were air dried and resuspended in 20–40 μL RNase free water.

To ensure quality of RNA samples, nanodrop measurements were taken and an A260/280 ratio of approximately 2 was considered pure. If readings were suboptimal ethanol precipitation with 3 volumes of 100% EtOH and 0.1 volume of 3 M sodium acetate at −20 °C for 16 h was performed to remove impurities and samples were centrifuged for 30 min at 13,000× *g* and 4 °C. This was followed by 2 washes of 500 μL of 75% EtOH and pellets were air dried and resuspended in 20 μL RNase free water. Samples had nanodrop measurements retaken to assess concentration and purity.

cDNA was prepared using Thermo Fisher (Waltham, MA, USA), Revert Aid First strand cDNA synthesis kit (K1621). Real time PCR was performed on all samples utilizing Qiagen’s RotorGene RT-PCR equipment (Qiagen, Germantown, MD, USA) and their RoterGene SYBR green PCR kit (Qiagen, Germantown, MD, USA, 204074) to amplify GAPDH, VE-cad and TMEM184A. Three biological replicates were completed in technical duplicate. Each sample had 200 ng of cDNA added. Cycling conditions included denaturation at 95 °C for 10 s and a combined annealing and extension for 60 s at 60 °C for 80 cycles followed with a melt curve to test specificity. Analysis of the data was calculated with the 2^-ΔΔCT method based upon GAPDH. Primers for both GAPDH and VE-cad were previously published by [34,35], respectively. and are shown in Table 4. The TMEM184A primers were designed by the authors:

### 2.10. VEGF Treatment and Vesicle Staining

VEGF-165 (Human VEGF 165 recombinant protein, Cat#48143 Cell Signaling, Danvers, MA, USA) treatments were administered to cells in culture in warmed media, 37 °C, 5% CO_2_ at 100 ng/mL for 5–20 min depending on the Rab-GTPase intracellular staining method (Rab4–10 min, and Rab11–20 min).

Cells treated with 10 or 20 min incubations of VEGF recombinant protein were harvested for IF treatment and fixed as previously described (see Immunofluorescence Staining), then incubated for 30 min in 0.5% Triton X-100 blocking and permeabilization buffer with constant rocking (adapted from [25]). Slips were incubated in primary and secondary antibody solutions, mounted in Vectashield, and imaged as described previously (see Immunofluorescence Staining). Images for VEGF treated cells show cells incubated in anti-Rab4 rabbit (1:50) and Alexa-647 anti-rabbit (1:200) with anti-TMEM184A NTD rat (1:50) and alexa-488 anti-rat (1:500) or show cells incubated in anti-Rab11a rabbit (1:50) and Alexa-647 anti-rabbit (1:200) with anti-TMEM184A NTD.

### 2.11. Corrected Total Cell Fluorescence (CTCF) and Image J Fiji Quantification

CTCF values for primary antibody staining of cell images were obtained using the lasso tool in Image J Fiji Win64 (2.9.0/1.54f; Java 18.0_172 (64 bit)) to measure the integrated density of each fluorophore and the background (area multiplied by the average mean density) was subtracted to correct for non-specific stain. CTCF values were normalized to the average of the CTCF control values for each protein measured to compare their relative absorbance values. Statistical significance between the siRNA control or mock control cells and siTMEM or TMEM OE cells was determined with one tailed, student t-tests. Protein levels in Western blotting were quantified using the Image J rectangular tool to plot a profile of their integrated densities. Densities were measured using the line tool to close peaks and the wand tool to highlight and measure peaks associated with protein bands. TMEM184A densities were normalized to that of Tubulin and VE-cad densities were normalized to that of Actin densities for quantitative comparison.

### 2.12. RStudio Visualization of Immunofluorescent Cell Experiments

RStudio (2024.04.2+764) software was used for visualization of siTMEM and TMEM OE static cell experiments. Multiple packages were utilized, including ggplot2, tidyr, readxl, dplyr, and scales to illustrate the density and distribution of the data. Tissue and vessel percent differences calculated in excel were input into RStudio to show varying titration concentrations and TMEM-Sdc4 synergy distributions. Cell CTCF data, sourced from IF images using Image J Fiji Win64 (2.9.0/1.54f; Java 18.0_172 (64 bit)), was normalized in Excel and reformatted in RStudio to compare TMEM184A and VE-cad CTCF values within siRNA control and siTMEM groups or mock control and TMEM OE groups. The creation of violin plots was performed using ggplot and geomviolin functions with plots customized with shades of red (TMEM184A) and green (VE-cad). For each respective plot, darker shades indicate control (siRNA), and lighter shades indicate knockdown (siTMEM). For OE plots compared to control plots the inverse is true, where lighter red and green violins represent control (mock) cell CTCF values and darker green and red violins represent TMEM OE CTCF values. Error bars were added to show measures of central tendency and variability using the scales package. Summary statistics, including mean and standard deviation, were calculated using data summary, and scales was employed for error bar placement and accuracy. These methods were utilized for clear visual representation of experimental and control cell data, allowing for effective comparison between knockdown and control TMEM184A and VE-cad in IF staining and of percent growth differences across fin injections. Violin plots were colorized for ZF data as described in the legend.

### 2.13. Scratch Wounding and Leading-Edge Analysis

BAOECs were transfected with either TMEM184A siRNA or control siRNA following our established electroporation protocol and maintained following established subculturing conditions. Cells were grown to >85% confluency then placed in a starvation media, containing only 0.2–0.5% Hi-FBS (Gibco, part of Thermo Fisher, Waltham, MA, USA), for one hour prior to scratching monolayers, allowing cells to acclimate to new culture media. Cells were then scratched (with a silicone spatula) and washed with one turn of 1X PBS before replacing fresh starvation media. All scratches occurred 24–48 h post electroporation. To calculate rate of closure, cells were imaged on a Nikon Eclipse TE2000-U microscope with a 10× objective at 0 and 8 h post scratch and processed with a Fiji Image J (2.9.0/1.53t; Java 1.8.0_322) plug-in [36]. Width of scratches at each timepoint were measured in μm and the rate of closure was calculated for 8 h post scratch using the following equation:

Rate (R) = (Width Initial (WI) − Width Final (WF))/(Time (T) in minutes)(1)

siTMEM and control siRNA calculated rates were normalized to the average rates of control siRNA per replicate. A Mann–Whitney U test was performed to determine significance (*p* < 0.05).

Cells were harvested at 0, 4, and 8 h for immunofluorescence visualization of the leading edge of scratch wounds using our established fixing and permeabilization protocol in 1X PBS 0.1% Triton-X 100 (above). Leading edges of the scratches were imaged using confocal microscopy (Zeiss) with a 63× objective, 1024 × 1024 acquisition, and 10–13 z-stacks. The scratch wound images were stained with 488 Phalloidin and mounted in Vectashield. Additional images were taken far away from each scratch as an internal control. The percentage of cells containing lamellipodia was calculated by noting if each cell on the leading edge of the scratch contained evidence of lamellipodia. A chi squared test was performed to determine significance (*p* < 0.05).

## 3. Results

### 3.1. TMEM184A Interacts with Sdc4 in Vascular ECs

TMEM184A is important for heparin-induced signaling in vascular cells, leading to decreased proliferation, and KD of TMEM184A decreases heparin-induced effects along with increasing the proliferation of vascular cells. Together this knowledge led us to hypothesize that endogenous HSPGs also interact with TMEM184A. We used a TMEM184A antibody from Invitrogen against the C-terminal domain (CTD antibody) (confirmed in BAOECs in Figure S1A–C) and a rat polyclonal antibody against an N-terminal bovine TMEM184A sequence (NTD) (designed by the authors and confirmed in Figure S1C, IF and Figure S1D, IP) to TMEM184A to facilitate these studies (See Section 2.4 for details). We noted that both TMEM184A CTD and NTD bind vascular cells in IF separately and when incubated together in a co-stain, TMEM184A NTD appears to outcompete the binding of TMEM184A CTD (Figure S1C), further supporting that both antibodies are binding regions of close proximity where TMEM184A is localized. TMEM184A NTD verification was further confirmed in a +NTD pull down in BAOEC lysate with TMEM184A CTD staining at 48 kDa and 55 kDa in WB (Figure S1D).

**Figure 1:**
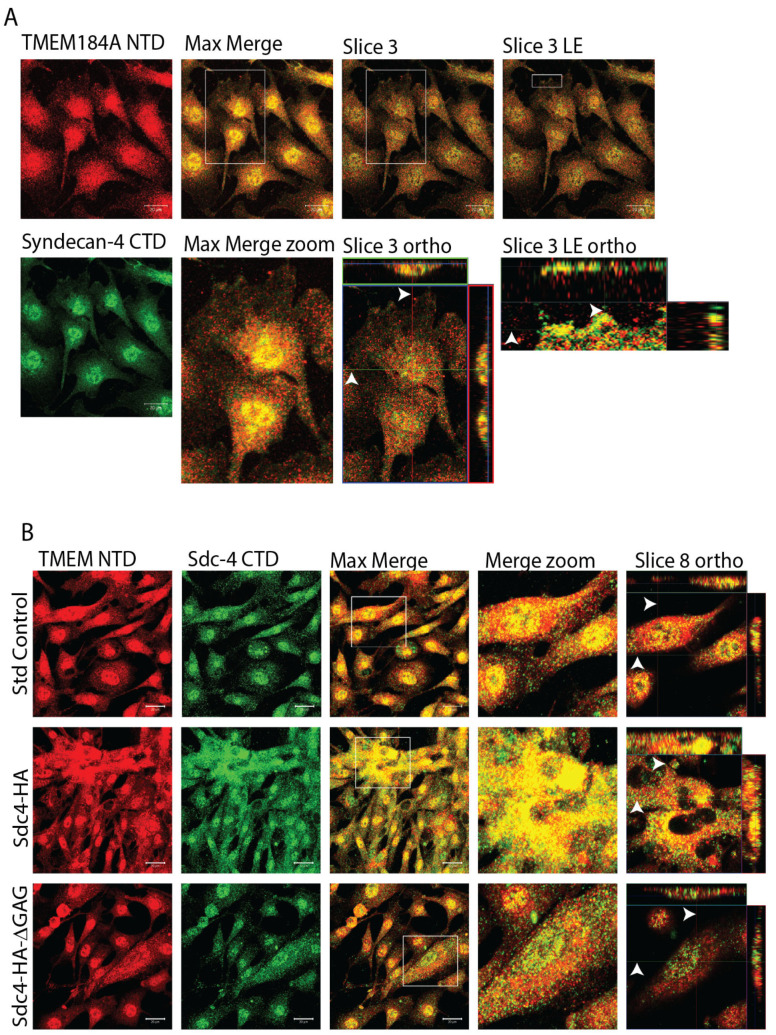
TMEM184A interacts with Sdc4 proteoglycans in BAOECs. (**A**) IF staining of TMEM184A NTD (red) merged with Sdc4 CTD (green) with maximum intensity (max, zoom projection) and slice 3 orthogonal (ortho) views of white boxes of slice 3 basal leading edge (LE). Z-position x and y coordinates and crosshairs correspond with white arrowheads in merged ortho images. Scale 20 μm, *N* = 18 across three independent experiments. (**B**) IF staining of lentiviral cell lines as described previously in A, with merged max intensity and slice 8 apical cell surface image projections (white boxes). Arrowheads denote x and y coordinates and crosshairs of the slice 8 z-position. Scale 20 μm. *N* = 6 for both Sdc4-HA and Sdc4-HA-ΔGAG transformations. *N* = 12 for standard control cells.

To examine interactions between TMEM184A and Sdc4, we first determined co-localization in cultured cells. Sdc4 co-localized with TMEM184A NTD and CTD domains in BOAECs (Figures 1A,B and S2A,B). Moreover, Sdc4 CTD and TMEM184A NTD appear to colocalize centrally within the cells and in vesical puncta that extend to the leading edge (LE) of the cell as well as the apical surface (Figure 1A orthogonal section zoom panels, white arrows denote the z position of the slices across the x and y orthogonal sections, respectively), while Sdc4 NTD and TMEM184A CTD appear to co-localize in perinuclear puncta that reach the apical surface (Figure S1A orthogonal section zoom panels). In initial assays with a 0.3% Triton-X 100 increased detergent percentage (Figure S2C), we observed lower levels of colocalization between both protein pairs overall, indicating that a semi-intact plasma membrane preserves these endogenous interactions.

In lentiviral BAOEC cell lines expressing Sdc4-HA, colocalization between TMEM184A and Sdc4 is markedly increased compared to standard control cells (Figure 1B first and second panel series) and appears to form diffuse aggregates at the apical surface (slice 8 ortho comparisons). However, we observed colocalization between Sdc4 and TMEM184A in Sdc4-HA-ΔGAG cells that closely resembled the events noted in standard control cells (Figure 1B third panel series), suggesting that the presence of HS chains promotes the interactions between the heparin receptor and the Sdc4 proteoglycan. Confirmation of Lentiviral HA constructs is illustrated in Figure S1E.

We evaluated these interactions with pull-downs of TMEM184A or Sdc4 shown in WB (Figures S1D and S2C), that co-precipitated the Sdc4 or the heparin receptor, respectively. This interaction appeared to increase with shorter incubation times and gentle agitation. However, these pull-downs were difficult to repeat consistently resulting in the antibody target protein loss as well as loss of partner. These data suggest that intra-cellular binding of the receptor and proteoglycans is potentially fleeting in vitro when cells are lysed to completion and confirm that processing of these complexes is problematic.

### 3.2. Sdc4 and Tmem184a Function Cooperatively to Promote Vessel and Tissue Outgrowth

**Figure 2:**
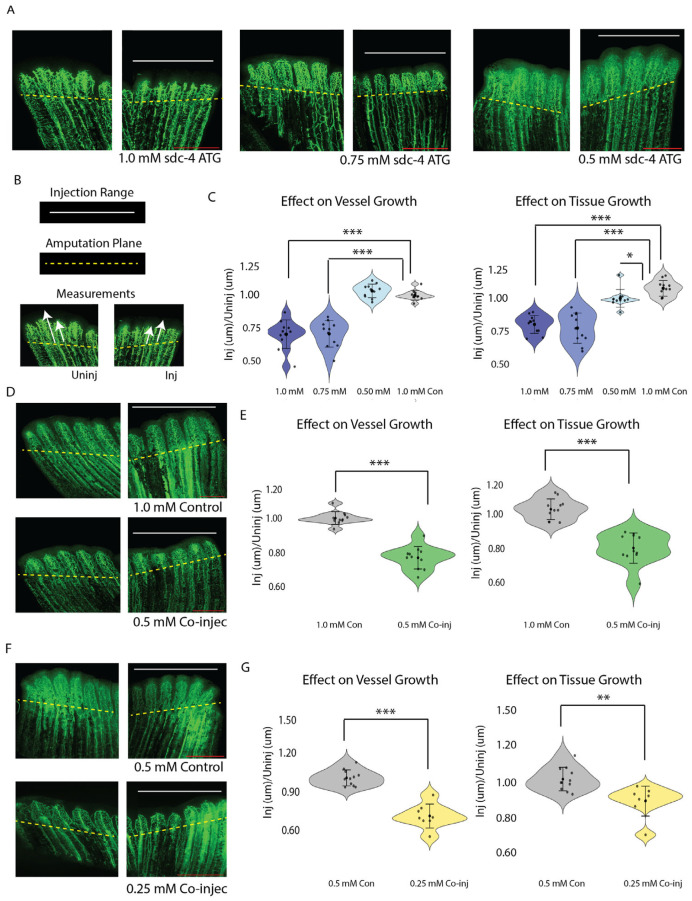
Combined subthreshold morpholinos *sdc4* and *tmem184a* blockers synergize to produce a vascular phenotype. (**A**) Representative confocal images of *sdc4* MO titration in *Tg(fli-egfp)* fin regenerants. Scale 500 μm. (**B**) Illustration of method showing injection range along fin rays, amputation plane demarcation, and measurements (denoted by white arrows) of vessel and tissue outgrowth comparison to generate percent difference calculations. (**C**) Violin plots of quantitation of s*dc4* MO titration injections (blue range violins from darkest (1.0 mM) to lightest (0.5 mM)) for vessel (**left**) and tissue (**right**) outgrowth of the uninjected and injected third rays compared with Lissamine control MO (gray violin) shown in A. s*dc4* blocker: *n* = 11 for 0.5 mM, 0.75 mM, and 1.0 mM representative groups, * *p* < 0.05 and *** *p* < 0.0005 in a student *t*-test. (**D**) Confocal images of *Tg(fli-egfp)* fin regenerants injected with 1.0 mM Lissamine control MO (1.0 mM Con) and combined 0.5 mM s*dc4* and 0.5 mM *tmem* morpholinos, (0.5 mM Co-injec). Scale 500 μm. (**E**) Violin plots of quantitation, as in C, showing direct comparisons of vessel and tissue outgrowth for the 1.0 mM Con (gray violins) and 0.5 mM co-injec (green violins) groups of the injected and uninjected third rays from the amputation planes, *n* = 11 for 1.0 mM Con and 0.5 mM Co-injec representative groups, *** *p* < 0.0005 in a student *t*-test. (**F**) Confocal images of *Tg(fli-egfp)* fin regenerants injected with 0.5 mM Lissamine control MO (0.5 mM Con) and combined 0.25 mM *sdc4* and 0.25 mM *tmem184a* ATG morpholinos, (0.25 mM Co-injec). Scale 500 μm. (**G**) Violin plots of quantitation, as in E, showing direct comparisons of vessel and tissue outgrowth for the 0.5 mM Con (gray violins) and 0.25 mM co-injec (yellow violins) groups of the injected and uninjected third rays from the amputation planes, *n* = 11 for 0.5 mM Con group and *n* = 8 for 0.5 mM Co-injec group, ** *p* < 0.005 in a student *t*-test, *** *p* < 0.0005.

Confirmation of Sdc4 and TMEM184A interactions in ECs, as well as recent literature findings that show Sdc4 requirement in pathological angiogenesis and wound healing [21,22], prompted us to consider whether both proteins cooperate in the same pathway to promote angiogenesis. In our previous work in ZF embryos, we observed no effect on ISV outgrowth with 0.5 mM *tmem184a* MO injections [29]. Here, we determined sub-threshold concentrations of *sdc4* MO by titrating 1.0, 0.75, 0.5 and 0.25 mM amounts, or injecting control MO at 1.0 mM and examining the phenotypes, comparing the outgrowth in MO injected half of the fin to the uninjected half. (Figure 2A,B). Percent difference calculations (as described in Methods) showed that vessel and tissue outgrowth were decreased by 20% in fins injected with a 1.0 mM–0.75 mM range of *sdc4* MO, while no significant decrease was observed with lower *sdc4* MO concentrations (* *p* < 0.05, *** *p* < 0.0005) (Figure 2B). To determine whether Sdc4 and Tmem184a synergize, we co-injected 0.5 mM *sdc4* MO and 0.5 mM *tmem184a* MO and compared the levels to 1.0 mM control MO and the subthreshold levels of each MO alone (Figure 2C). Percent difference calculations indicated 20% decreased outgrowth in the co-injected fins (*** *p* < 0.0005) (Figure 2C). We also examined subthreshold injections at 0.25 mM concentrations for both MOs, as well as 0.5 mM control MO (Figure 2D). Our statistical analysis of fins injected with half of the subthreshold concentrations of both morpholinos in a co-injection confirmed the cooperation of both proteins in vessel (*** *p* < 0.0005) and tissue repair (** *p* < 0.005), which suggests the interaction is critical for reparative angiogenesis in wound healing.

### 3.3. TMEM184A Is Required to Maintain Post-Translational VE-Cad Levels in Sub-Confluent ECs

Our recent developmental angiogenesis study in ZF found that Tmem184a KD significantly decreased levels of total VE-cad in embryo lysates and in developing ISVs, in parallel to decreased angiogenesis [29]. Coupled with published evidence of Sdc4 involvement in EC migration and VE-cad recycling and our TMEM184A-Sdc4 interactions above, these findings led us to ask whether the stability of VE-cad was dependent on TMEM184A expression and function in cultured ECs.

**Figure 3:**
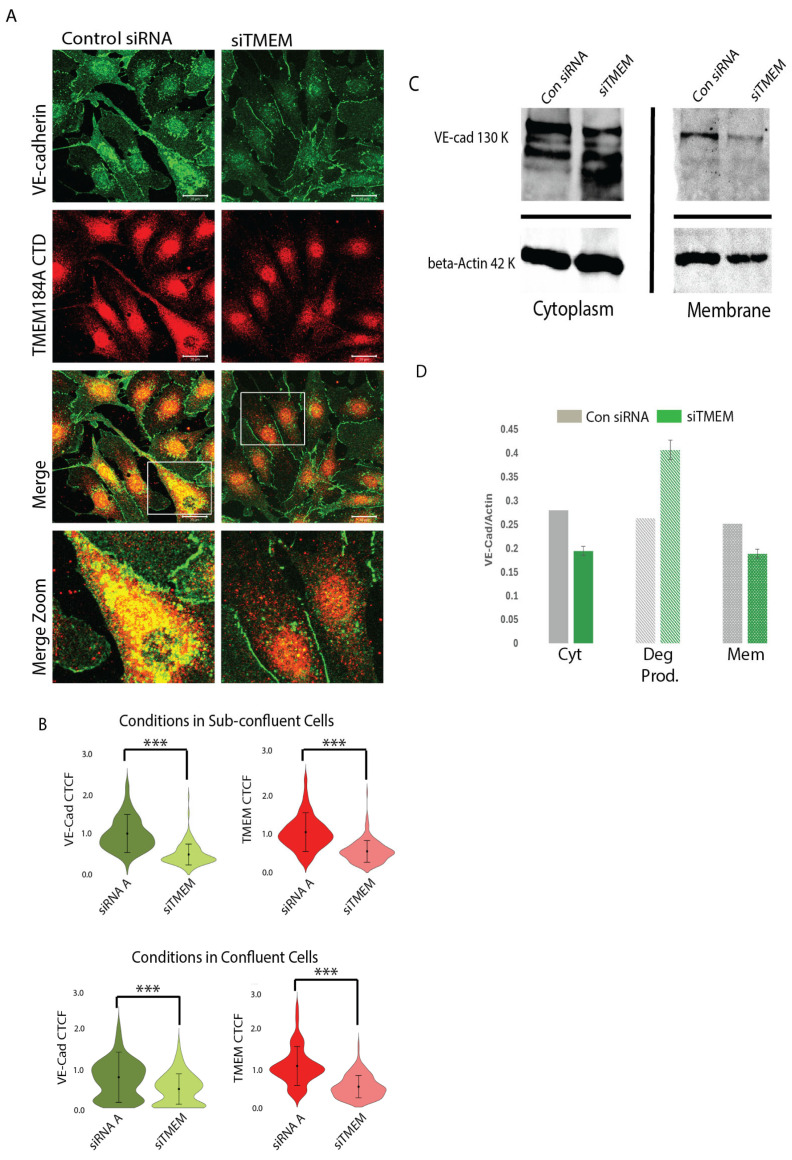
TMEM184A siRNA KD decreases post-translational levels of VE-cad in the membrane and in cytoplasmic vesicles. (**A**) Comparisons of VE-cad (green) and TMEM CTD staining (red) in control siRNA (Con siRNA) and siTMEM BAOECs. Reduced levels of VE-cad and TMEM are highlighted in merged, white box, zoom, comparisons. Scale 20 μm. (**B**) Violin plots of corrected total cell fluorescence (CTCF) values of VE-cad and TMEM184A across Con siRNA (dark green and dark red violins) and siTMEM cells (light green and light red violins) in sub-confluent and confluent BAOECs across three independent experiments, *n* = 179 in sub-confluent condition, *n* = 193 in confluent condition, *** *p* < 0.0001. (**C**) WB of Con siRNA and siTMEM subcellular fractionation lysates comparing Cyt (supernatant) and Mem (pellet) fractions. Cyt fractions show full length VE-cad (130 kDa) and Actin loading control (42 kDa) with increased degradation in the siTMEM lane. In Mem fractions, full length VE-cad and Actin loading control bands are compared in one independent experiment. (**D**) Quantification of WB of subcellular fractionation densities of VE-cad normalized to Actin for full length VE-cad, VE-cad degradation products, and membrane VE-cad bands from one representative blot.

We employed a transient transfection of BAOECs with bovine TMEM184A siRNA and compared VE-cad antibody staining in IF with that of siRNA control cell staining. Our analysis of stained transfected cells confirmed transient TMEM184A KD in cells with 65–75% confluence (Figure 3A) and in cells with confluence greater than 90% (IF images shown in Figure S3B and quantitation in Figure 3B) through a calculation of the relative absorbance of the TMEM184A siRNA compared to control siRNA cells (Figure 3B). Relative absorbance of VE-cad staining in the TMEM184A KD sub-confluent cells compared to control cells decreased by about 50–60%, (Figure 3B). Visually, the greater effect of TMEM184A siRNA on sub-confluent cells shows a marked decrease in VE-cad puncta when compared directly to TMEM184A siRNA knockdown VE-cad staining (Figure 3A, boxed and zoomed images), indicating that TMEM184A KD significantly affects the trafficking and sorting of VE-cad in actively proliferating and migrating cells.

To determine whether VE-cad membrane stability was affected in KD cells we fractionated TMEM184A siRNA and control siRNA cells into cytoplasmic (supernatant) and membrane (pellet) samples and evaluated their VE-cad levels with WB (see Materials and Methods for details). This protocol employed a moderately fast (13,000× g) and long (12 min) cold spin that efficiently separated all cytoplasmic components including vesicles and intra organelle components from the membranes of the cell including the plasma, ER and nuclear membranes. In WB of sub-confluent cells (Figure 3C), both cytoplasm (Cyt) and membrane (Mem) VE-cad levels are decreased in TMEM184A siRNA (siTMEM) cells compared to siRNA control cell levels and siTMEM cells show increased VE-cad degradation (Deg Prod.) compared to control cells. (Figure 3D)

RT-qPCR of siTMEM184A and siRNA control sub-confluent cell populations showed confirmation of decreased TMEM184A transcript in siTMEM cells normalized to GAPDH (0.12-fold). Relative levels of VE-cad transcript neither increased or decreased relative to TMEM184A or GAPDH transcript levels, suggesting that VE-cad transcriptional changes are not affected by changes in TMEM184A expression. Taken together with our IF and WB results, these data support that TMEM184A is required to maintain post-translational VE-cad levels and VE-cad membrane stability, potentially through the regulation of its sorting and trafficking.

### 3.4. TMEM184A-tGFP Expression Colocalizes in VE-Cad Puncta and Increases Total VE-Cad in Sub-Confluent ECs

**Figure 4:**
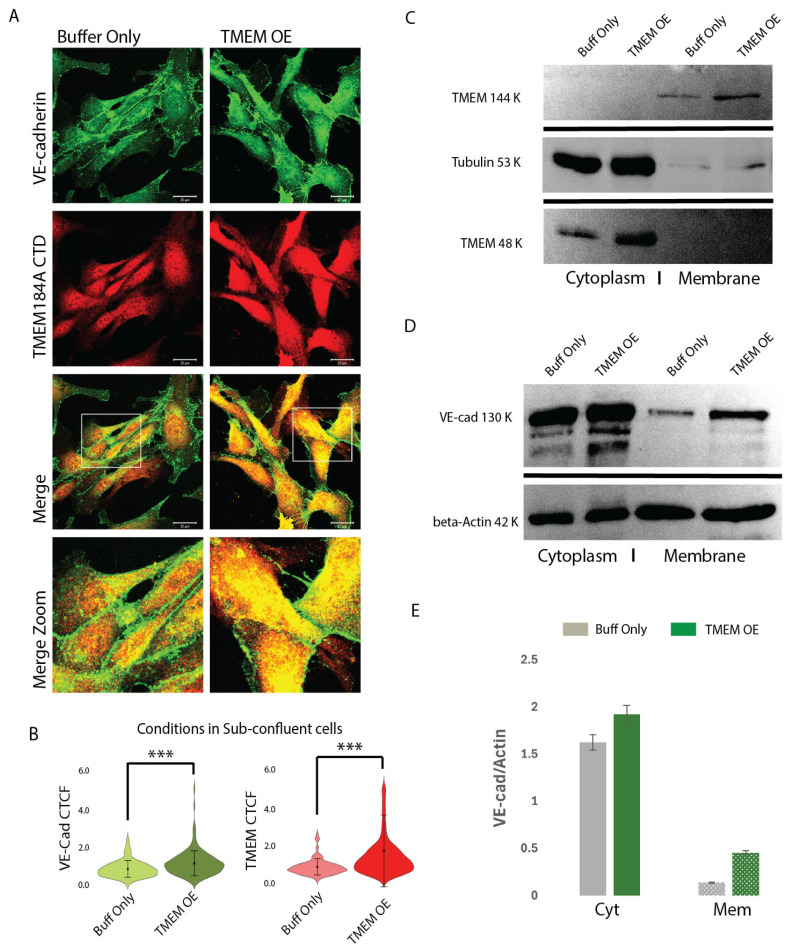
TMEM OE increases VE-cad rapid recycling and membrane levels. (**A**) Comparisons of VE-cad (green) and TMEM184A CTD (red) staining in mock control and TMEM OE BAOECs. Increased levels of VE-cad and TMEM and VE-cad-TMEM colocalization in TMEM OE are highlighted in merged, white box, zoom, comparisons. Scale 20 μm. (**B**) Violin plots of corrected total cell fluorescence (CTCF) values of VE-cad and TMEM184A across cells electroporated with buffer only (buff only, light green, and light red violins) and cells electroporated with TMEM-tGFP (TMEM OE, dark green and dark red violins) in sub-confluent BAOECs across three independent experiments, *n* = 198, *** *p* < 0.0001. (**C**) TMEM OE confirmation with TMEM184A CTD staining in cells electroporated with buffer only (Buff only) and cells electroporated with TMEM-tGFP showing TMEM184A CTD (144 kDa, 48 kDa) and Tubulin (53 kDa). (**D**) Representative WB of subcellular fractionation samples from buff only and TMEM OE cells, VE-cad (130 kDa), beta-actin (42 kDa). WB was obtained in duplicate, once with beta-Actin and once with Tubulin. (**E**) Western blot quantifications of VE-Cad densities normalized to actin for Cyt and Mem fractions of buff only cells (gray boxes) and TMEM OE cells (green boxes) from the representative blot shown in (**D**).

Since TMEM184A siRNA significantly decreased VE-cad levels in cell culture, we asked whether overexpression of the TMEM184A receptor would increase VE-cad levels. We used electroporation of a TMEM184A-tGFP construct compared with a mock control electroporation in buffer only. TMEM184A-tGFP expression (TMEM OE) showed increased colocalization rates of TMEM184A with VE-cad in both vesicles and perinuclear regions when compared to mock electroporation (Figure 4A, boxed and zoomed images) and increased relative absorbance of both VE-cad and TMEM184A were observed in TMEM OE compared to mock control cells (Figure 4B). This was also visually observed in the increased density of VE-cad in AJ sites in static TMEM OE cells compared with mock control cells. To confirm TMEM OE electroporation as well as IF findings, and to determine whether TMEM OE increases VE-cad membrane stability, we used subcellular fractionation and immunostaining of TMEM184A and VE-cad in WB to show differences in the relative densities of Cyt (supernatant) and Mem (pellet) samples (Figure 4C,D). Fractionated samples of TMEM OE showed increased levels of TMEM184A CTD in both cytoplasm and membrane fractions (Figure 4C), as well as increased stability of VE-cad in the membrane, with an increased amount of VE-cad and VE-cad degradation products in the Cyt of TMEM OE cells (Figure 4D). Quantitation of Cyt and Mem VE-cad bands shown in Figure 4D confirmed an increase in the relative densities of VE-cad bands normalized to Actin in TMEM OE cells compared to mock control cells (Figure 4E). Collectively, these results suggest that constitutive recycling of TMEM184A is induced in TMEM OE cells and results in increased recycling and membrane recovery of VE-cad.

### 3.5. TMEM184A Colocalizes with Rab-GTPases in Response to VEGF

**Figure 5:**
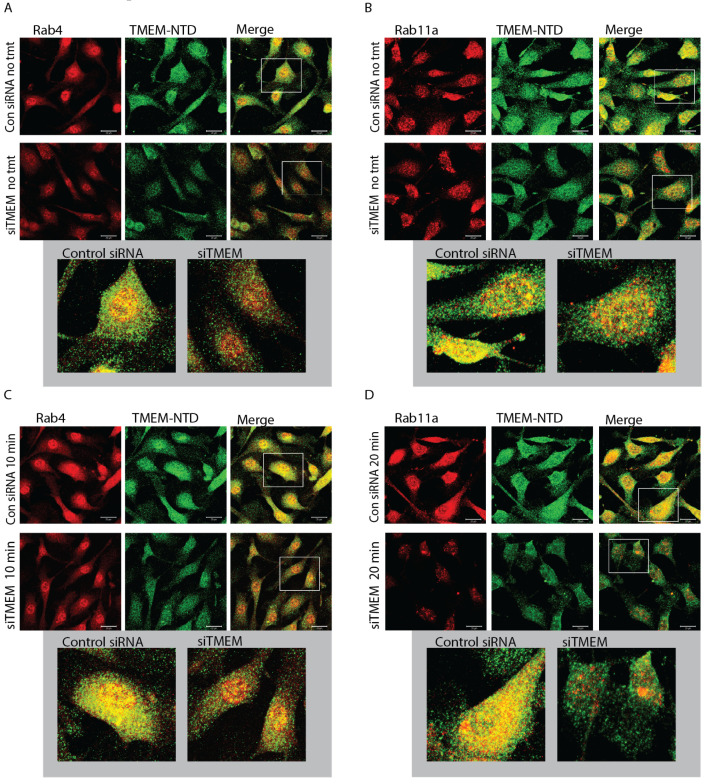
TMEM184A colocalizes with recycling Rab-GTPases in the presence of VEGF. BAOECs were treated with VEGF-165 or left untreated as noted, fixed, and permeabilized with 0.5% Triton X-100 to increase the visibility of vesicles. (**A**) In untreated cells (no tmt), Rab4 (red) colocalizes with TMEM184A NTD (green) in bright yellow puncta (gray box, max intensity projection zoom). Scale 20 μm. Images are representative of 19 Con siRNA images and 17 siTMEM images from four independent experiments. (**B**) Rab11a (red) colocalizes with TMEM184A NTD (green) in bright yellow and orange puncta (gray box, max intensity projection zoom) in cells without treatment. Scale 20 μm. Images are representative of 11 Control and KD images from two independent experiments. (**C**) Rab 4 (red), (**D**) Rab11 (red) and TMEM184A NTD (green) colocalization appears to increase (gray boxes) upon 10 and 20 min VEGF treatment. Scale 20 μm. Images are representative of 13 Con siRNA images and 11 siTMEM images from two independent experiments for C., and 11 images for Control and KD cells across two independent experiments for D.

Because TMEM184A forms vesicle puncta, VE-cad levels were significantly decreased in siTMEM ECs both at adhesion sites and in intracellular puncta, and TMEM OE cells exhibited increased vesicles of TMEM184A colocalized with VE-cad, we asked whether TMEM184A colocalizes with Rab4 or Rab11a, key trafficking players in ECs with induced growth factor signaling through VEGF treatment. In IF staining of untreated BAOECs, TMEM184A colocalizes with Rab4 in puncta perinuclearly (Figure 5A, gray box, zoom), but in few cytoplasmic vesicles and there is a visible decrease in Rab4 fluorescence levels and colocalization with TMEM in untreated siTMEM cells. In untreated BAOECs, we also observed greater colocalization of Rab11a with TMEM in bright perinuclear yellow and orange puncta compared to Rab4 interactions (Figure 5B, gray box, zoom), and Rab11a vesicles and colocalization events were visibly decreased in siTMEM cells. In BAOECs treated with 10 min of VEGF, TMEM184A and Rab4 colocalization in vesicles is visibly increased in control cells (Figure 5C, gray box, zoom) while Rab4 fluorescence levels increase and colocalize with TMEM to a lesser extent in treated siTMEM cells. In cells treated with VEGF for 20 min, we observed increased Rab11a and TMEM184A colocalization in diffuse yellow and orange vesicles (Figure 5D, gray box, zoom), while siTMEM cells maintain low levels of Rab11a and very little colocalization compared to that of control cells with 20 min VEGF treatments. Taken together, these data suggest that TMEM184A is a component of the sorting and late endosomal trafficking system in vascular ECs in response to VEGF.

### 3.6. TMEM184A KD Cells Migrate Faster Compared to Control siRNA Cells in Wounding

**Figure 6:**
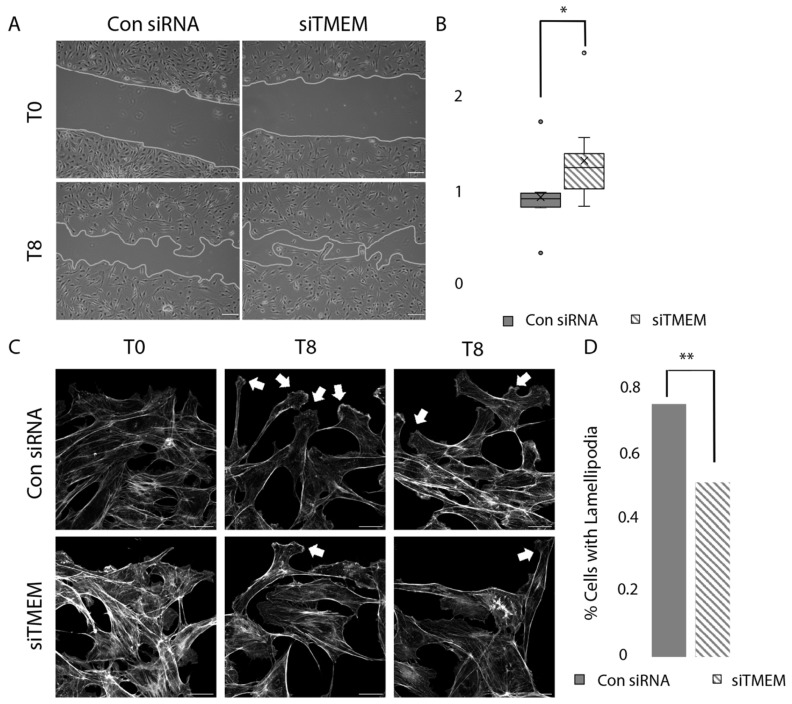
Rate of closure in siTMEM and siRNA cells. (**A**) Phase contrast (10×) images of transiently transfected BAOEC (siTMEM or control siRNA) cells undergoing migration 0 and 8 h post scratch. Seeded on 0.2% porcine gel; conducted in 0.2–0.5% Hi-FBS MEM media, 37 °C, and 5% CO_2_. The gray line indicates the leading edge of migration. Scale 100 μm. (**B**) Rate of closure per well (siTMEM *n* = 11, control siRNA *n* = 10) was calculated and normalized to average rate of closure for siRNA per replicate (*N* = 3). A Mann–Whitney U test was performed, * *p* < 0.05. (U = 21, *p* = 0.016). (**C**) Confocal microscopy of the leading edge at 0 and 8 h post scratch (Gray: Phalloidin) (scale 20 μm). White arrows indicate lamellipodia during cell migrating at the leading edge at T8. (**D**) Percentage of migratory front cells containing lamellipodia (KD *n* = 125, control siRNA *n* = 132 cells). A chi-squared test was performed ** *p* < 0.001.

Previously, we had shown that Tmem184a KD in the regenerating ZF caudal fin resulted in aberrant vascular outgrowth [30]. Since TMEM184A KD also decreases VE-cad levels in cells, we hypothesized that TMEM184A KD would alter the speed and/or organization of migration in cultured ECs. We sought to confirm this finding through determining whether TMEM184A siRNA treated ECs migrate differently compared to control siRNA cells in scratch wound healing assays. Upon examining scratch wound areas 8 h post wounding, we observed that siTMEM cells migrated faster than control siRNA cells at 8 h post scratch (Figure 6A). To quantify this finding, the relative rate of closure was determined for siTMEM and control cells over 8 h post wounding. Migration of cells into the wound area during the eight hours was significantly faster in siTMEM cells (Figure 6B). Following our brightfield observations of cell migration, we examined the wounded layers for morphological differences in migrating cells by looking at the filamentous actin in cells at the leading edge of the scratch (Figure 6C). We determined that the KD cells contained approximately 30% fewer lamellipodia compared to the control cells at 8 h post scratch (Figure 6D). Taken together, these data suggest that TMEM184A expression is required for consistent directional movement in collective cell migration in wound healing.

## 4. Discussion

Our early experiments with cultured vascular cells indicated that heparin treatment induced MAPK signaling changes downstream of PKG activation and increased c-GMP production [2,6]. We now know that heparin is a ligand that binds TMEM184A and that TMEM184A is required for at least some of the downstream heparin effects we observe in vascular cells, including decreased levels of p-ERK in VSMCs, and DUSP1 dependent decreased levels of p-p38 and p-JNK as well as decreased levels of stress fiber formation in the presence of inflammatory mediators in ECs [1,4,6,37]. Moreover, the decreased p-ERK antiproliferative effect specific to the vasculature also requires calcium dependent eNOS activation in focal adhesion (FA) sites [3]. Our characterization of TMEM184A in studies in vivo, has shown that Tmem184a is required for proper vascular regeneration in the ZF caudal fin in a manner that slows proliferation, potentially impacting cell organization and polarity [30]. In developing ZF embryos, *tmem184a* MO KD produced similar vascular defects [29].

In several pathological angiogenesis mouse models, Sdc-4 interacted with VE-cad in an HS independent manner and was required for VE-cad internalization and processive angiogenesis [21]. VE-cad is a mechanosensory protein that is integral to AJs and forms homotypic associations in clusters with VE-cad in the membrane of neighboring cells. These junctions involve numerous other proteins, allowing the endothelium to respond to blood flow and vascular damage [38,39]. There is additional supporting evidence in the literature that Syndecans play crucial roles in the localization and trafficking regulation of cell surface mechanosensors, including the trafficking of Cadherin and Integrin types to stabilize AJs and FAs in migrating fibroblasts [18,40].

In the present study, we examined the hypothesis that TMEM184A interacts with Sdc4 in vivo. In cultured ECs, we employed antibodies against both the N-terminal and C-terminal domains of TMEM184A (previously identified to be on the extracellular surface of the plasma membrane [4]). We found co-localization between TMEM184A and Sdc4 at the leading edge of cells spreading and in distinct puncta on the basal and apical surfaces of cells that markedly increased in Sdc4 OE cells. This effect was not observed in cells overexpressing Sdc-4 without the HS chains (Sdc4-HA-∆GAG), suggesting that HS chains promote this interaction. Both antibodies against the extracellular domain (N-terminal region) and cytoplasmic domain (C-terminal region) of Sdc4 appear to co-localize with TMEM184A (Figure S2A,B), and co-localization appears to be stronger when lower detergent concentrations are employed to preserve plasma membrane components (Figure S2B,C), though orthogonal sections also indicate co-localization in the cytoplasm (Figure S2A). In addition, Sdc4 and Tmem184a synergize in a zebrafish caudal fin regeneration model supporting the idea that these two proteins interact in vascular function. It is likely that other HSPGs can also interact with TMEM184A through their HS chains (or possibly also through protein–protein interactions).

The requirements for Sdc4 in migration may be critical for wound healing [41]. Homozygous and heterozygous Sdc4 KO in an epithelial mouse model showed decreased size in the developing microvasculature of epidermal granular tissue, and Sdc4 KD in HUVECs slowed vascular tube formation and decreased cortical actin fiber rings and vinculin puncta, promoting cell elongation and decreasing cell roundness [16,22]. In a related study, Sdc4 inhibition of Rac activity promoted directional cell migration in neural crest cells [19]. These data suggest that while Sdc4 is required for internalization of VE-cad in order that AJs may decouple and reform in cell proliferation and migration [21], forces exerted directly or indirectly through flow, or the ECM also impact VE-cad movement and junctional integrity. Collectively, these data support a model in which TMEM184A-Syndecan interactions may impact mechanosensory signaling that promotes the polarity and cytoskeletal organization of moving cells through the regulation of mechanosensory trafficking [42,43].

In addition to the observation that Tmem184a KD induced truncation of ISVs outgrowth in the developing ZF embryo, Tmem184a KD reduced total VE-cad levels in proliferating stalk cells, suggesting that TMEM184A expression and or function is required to maintain VE-cad levels in proliferating cells [29]. In studies of VE-cad in static cells and in flow, Src family kinases and vascular endothelial-protein tyrosine phosphatase (VE-PTP) contribute to the phosphorylation schemes of the cytoplasmic tail of VE-cad, its stability in the membrane, and its rapid recycling or degradation in the remodeling of AJs, reviewed in [44]. In a recent study of VE-cad in flow, activated Src-family related YES kinase at the plasma membrane, was required for VE-cad Tyr phosphorylation and internalization in HUVECs and vascular tissues [45], and YES deletion increased cortical actin ring bundles and collective cell migration rates in HUVECs and promoted leakage in a vascular mouse model [45].

Here, we find that depletion of TMEM184A in proliferating BAOECs in culture decreases the relative levels of VE-cad in AJs and in intracellular vesicles without decreasing relative mRNA levels. This effect is observed with statistical significance, but to a lesser extent, in siTMEM confluent monolayers, suggesting that the recovery of VE-cad in proliferating and migrating cells and in junctional remodeling of monolayers is dependent at least in part on TMEM184A expression. Conversely, we found that TMEM184A OE sub-confluent ECs displayed relatively increased levels of VE-cad that colocalized with TMEM184A, leading to jagged AJs and rounded morphology likely due to increased VE-cad membrane turnover.

VE-PTP dynamics have been shown to classically regulate AJ dynamics through dephosphorylation mechanisms at the CTD of VE-cad that either promote p120-catenin binding and its membrane stability or VE-cad internalization to early endosomes [46,47]. VE-PTP was also shown to prevent VE-cad internalization and promote junctional integrity through binding of RhoGEF GEF-H1, sequestering it from Rho and reducing Rho activation at AJs [48]. Our study of inflammatory signaling in ECs showed that TMEM184A was required for the induction of dual specificity phosphatase-1 (DUSP1) in the presence of heparin, and that this event reduced p-p38 and pJNK levels in modulation of the inflammatory response [1]. Since TMEM activation with heparin upregulated DUSP1, the possibility that TMEM expression and function increases PTP cell surface activation or Rho activity suggests further investigation.

Similarly to our findings in the ZF caudal fin, developing embryo, and in vascular cell culture, CMTM4 MO KD in ZF also truncated ISV outgrowth, and OE of CMTM4 in HUVECS enhanced vesicular VE-cad and increased Rab4, Rab11, and Rab7 GTPases promoting both degradation and turnover as well as rapid recycling of VE-cad to AJs [27]. Both the finding that CMTM4 colocalized with Rabs in its upregulation and increased VE-cad turnover and the finding in this study that TMEM OE increased vesicle puncta that contained both TMEM184A and VE-cad, prompted us to investigate whether TMEM184A colocalized with Rab-GTPases. This question is further supported by earlier findings in published literature of TMEM184A (Sdmg-1) where Sdmg1 is observed colocalized with vascular associated membrane protein-1 in non-vascular cell types [28,49]. Indeed, we found that TMEM184A colocalizes with Rab4 vesicles and Rab11a endosomes, indicating that TMEM184A promotes VE-cad vesicle transport recovery to AJs in proliferating ECs. We also observed an increased co-localization of TMEM184A with Rab4 and Rab11 in VEGF treated control cells, though this is less apparent in TMEM184A KD cells. Significant evidence supports Rab11a involvement in VE-cad recycling [50]. The fact that Rab11a decreases results in reduced VE-cad recycling and vesicle leakage [51], suggesting that the apparent Rab11a decreases we note in TMEM184A KD cells (Figure 5) might link this Rab activity to TMEM184A function.

ECM changes, such as increased stiffness or the deposition of Fibronectin in the matrix, recruit and activate scaffolding players that promote specific Integrins to the membrane and regulate focal Adhesion (FA) stability [52,53]. Rac and Rho signaling regulates cortical actin bundles and actomyosin pulling forces on stress and intermediate fibers, respectively, that further remodel AJs and FAs, and Sdc4 colocalizes with FAK and is required for the maintenance of specific Cadherins in fibroblast AJs in an HS dependent manner [9,40]. Since Sdc4 directly impacts migratory behavior in cells through interactions with fibronectin ECM and through the regulation of Integrin recruitment to FAs [18], we asked whether the vascular phenotype that resulted from Tmem184a and Sdc4 subthreshold MO injections were due in part to changes in cell migration.

Studies of Sdc4 mediated force transduction that recruits talin-1 and kindlin-2 to FAs to the basement membrane shows that propagated signal requires ROCK1 and PI3K, like that of signal transduction induced through forces applied to VE-cad; and Sdc4 Fibronectin complex formation induces Caveolin-1 dependent Rac1 endocytosis that promotes filopodia extension and directional migration in fibroblasts [20,54]. Since Integrin and VE-cad recycling dynamics and localization to the membrane require dynamic regulation for actin cytoskeletal organization and directional migration [23,24,55] and also require Sdc4 and Syntenin binding [18,56], we asked whether siTMEM cells exhibit migratory defects in scratch wound healing assays. Our results demonstrated that siTMEM cells migrated at a faster rate compared with control cells but in a less coordinated fashion. This finding is further supported by visualization of the leading edge of migrating cells where lamellipodia are significantly decreased in siTMEM cells. Further, in our recent studies in cells, TMEM184A activation through heparin treatment resulted in TMEM184A colocalization with eNOS in FAs, an outcome that required signaling through transient receptor potential vanilloid-type 4 (TRPV4) and eNOS activation [3]. Flow also leads to TRPV4 clustering at FAs [57,58], while TRPV4 KD in ECs leads to decreased VE-cad at AJs [59], indicating a link between migration and cell-adhesion that may involve TMEM184A. Collectively, our findings and published data support a mechanism in which a loss of Sdc4-TMEM184A interactions and/or VE-cad recovery at the membrane surface in siTMEM cells decrease lamellipodia formation through changes in VE-cad recruitment and Actin polymerization.

This study of the novel heparin receptor, TMEM184A, has provided new knowledge in its characterization of a dual function of the receptor in signaling and trafficking that is required to maintain vascular integrity in wound healing and immune responses as a direct regulator of junctional AJ dynamics through VE-cad turnover. E-cadherin binding induced intracellularly through Cadherin–Catenin Clusters (CCCs) driven by Catenin Associated Proteins (CAPs) forming super-complex arrangements outside of traditional AJs formed through Cadherin homotypic associations are a phenomenon observed specifically in epidermal cells [60]. However, this unusual finding fits a model where dual signaling/recycling proteins such as TMEM184A help to remodel AJs through recruitment of scaffolding proteins regulated by intercellular signaling and trafficking that synchronizes signals propagated across cell monolayers and through tissues. A recent study of a shear stress model in HUVECs showed that NRP-1 localization to AJs stabilized VE-cad in the membrane and promoted p120-Catenin binding promoting cytoskeletal realignment in flow [61], yet another potential mechanism in which TMEM184A signaling and recycling may promote VE-cad stability in endothelial remodeling.

## 5. Conclusions

Here, we have presented strong evidence that TMEM184A has a dual function in vascular cells through signaling and trafficking mechanisms. Due to progress in clinical cardiovascular research, a broader range of risk factors associated with atherosclerosis, including inherited genetic variants and chronic inflammatory diseases, are now known. These factors present a critical need to develop treatments that reduce cardiovascular inflammation that may lead to atherosclerosis and major cardiac events. Elucidating TMEM184A’s role specific to the regulation of permeability, cell proliferation, and migration in vascular cells will provide us with new knowledge required to develop therapeutic strategies that target TMEM184A to slow the progression of chronic inflammation in atherosclerosis.

## Figures and Tables

**Table 1:** Primary and Secondary antibodies used in the study.

Primaries	Host Organism	Company	Catalog Number	RRID
Sdc4	Rabbit	BioVision	Cat# 3644-100	AB_2183016
Sdc4	Mouse	Santa Cruz Biotechnology	Cat# sc-12766	AB_628314
TMEM184A	Rabbit	Thermo Fisher Scientific	Cat# PA5-96834	AB_2808636
TMEM184A	Rat	GenScript	protein G-purified peptide sequence from the N-terminal region of bovine TMEM184A (N-PAGPQMDHMGNSSQC)	
VE-cadherin	Goat	Santa Cruz Biotechnology	Cat# sc-6458	AB_2077955
VE-cadherin	Rabbit	Cell Signaling Technology	Cat# 2500	AB_10839118
Rab4	Rabbit	Abcam	Cat# ab13252	AB_2269374
Rab11a	Rabbit	Cell Signaling Technology	Cat# 5589	AB_10693925
HA	Goat	Novus	Cat# NB600362	AB_10124937
β-Actin	Rabbit	Cell Signaling Technology	Cat# 4970	AB_2223172
Tubulin	Mouse	Abcam	Cat# 7291	AB_2241126
**Secondaries**	**Host Species**	**Company**	**Catalog number**	**RRID**
Alexa 647 anti-mouse	Donkey	Jackson ImmunoResearch Labs	Cat# 715-605-151	AB_2340863
Alexa 647 anti-Rabbit	Donkey	Jackson ImmunoResearch Labs	Cat# 711-605-152	AB_2492288
Alexa 647 anti-rabbit, FC specific	Goat	Jackson ImmunoResearch Labs	Cat# 111-605-046	AB_2338076
Alexa 488 anti-rat	Donkey	Jackson ImmunoResearch Labs	Cat# 712-545-153	AB_2340684
Alexa 488 anti-rat, F(ab’)_2_ specific	Donkey	Jackson ImmunoResearch Labs	Cat# 112-545-072	AB_2338359
Alexa 488 anti-rabbit	Donkey	Jackson ImmunoResearch Labs	Cat# 711-545-152	AB_2313584
Alexa 488 anti-goat	Donkey	Jackson ImmunoResearch Labs	Cat# 705-545-147	AB_2336933
CY3 anti-mouse	Donkey	Jackson ImmunoResearch Labs	Cat# 715-165-150	AB_2340813
TRITC anti-rabbit	Donkey	Jackson ImmunoResearch Labs	Cat# 711-025-152	AB_2340588
TRITC anti-goat	Donkey	Jackson ImmunoResearch Labs	Cat# 705-025-147	AB_2340389

**Table 2:** Primary Antibody use in Immunofluorescence.

Figure	Primary1	Primary2	Secondary1	Secondary2
Figure 1A,B	Sdc4 rb (1:100)	TMEM NTD rat (1:50)	Alexa 647 α-rb (1:200)	Alexa 488 α-rat (1:500)
Figure S1C	TMEM CTD rb (1:50)	TMEM NTD rat (1:50)	TRITC α-rb (1:200)	Alexa 488 α-rat (1:500)
Figure S2C	Sdc4 rb (1:100)	TMEM NTD rat (1:50)	Alexa 647 α-rb (1:200)	Alexa 488 α-rat (1:500)
Figure S2A,B	Sdc4 mo (1:50)	TMEM CTD rb (1:50)	Alexa 488 α-mo (1:200)	TRITC α-rb (1:200)
Figure 3 and Figure 4	VE cad gt(1:200)	TMEM CTD (1:100)	TRITC α-gt (1:200)	Alexa 647 α-rb (1:200)
Figure S3	VE cad gt (1:200)	TMEM CTD (1:100)	Alexa 488 α-gt (1:200)	Alexa 647 α-rb (1:200)

**Table 3:** Western Blot antibody use.

Figure WB	Primary1	Primary2	Secondary1	Secondary2
Figures 3C and 4D	VE-cad rb (1:1000)	β-actin rb (1:1000)	Alexa-488 α-rb (1:10,000)	Alexa-488 α-rat F(ab’)_2_ specific (1:10,000)
Figure 4C	TMEM CTD rb (1:500)	Tubulin mo (1:10,000)	Alexa-488 α-rb (1:10,000)	Alexa 647 α-mo (1:10,000)
Figure S1A,B	TMEM CTD rb (1:500)	Tubulin mo (1:10,000)	Alexa-488 α-rb (1:10,000)	CY3 α- mo (1:10,000)
Figure S1D	TMEM CTD rb (1:200)	Sdc4 mo (1:200)	Alexa 647 α-rb (1:10,000)	CY3 α- mo (1:10,000)
Figure S1E	HA gt (1:1000)	Tubulin mo (1:10,000)	Alexa-488 α-gt (1:2000)	CY3 α- mo (1:10,000)
Figure S2D	Sdc4 rb (1:500)	TMEM NTD rat (1:200)	Alexa 647 α-rb FC site Specific (1:10,000)	Alexa-488 α-rat F(ab’)_2_ specific (1:10,000)
Figure S3A	VE-cad goat (1:5000)	Tubulin mo (1:10,000)	Alexa-488 α-goat (1:10,000)	Alexa 647 α-mo (1:10,000)

**Table 4:** PCR Primers.

Primer Name	Primer 5′-3′	Reference
GAPDH Forward	ACACCCTCAAGATTGTCAGCAA	[34]
GAPDH Reverse	TCATAAGTCCCTCCACGATGC	[34]
VE-Cadherin Forward	TCTGCCGGCAAGGTGTTCCG	[35]
VE-Cadherin Reverse	CATGGTCTGCCACCGTGGGG	[35]
TMEM184a Forward	CTTCTGCAAGCAGCCCAC	
TMEM184a Reverse	CCTGAAGTTGCAGGCGTC	

## Data Availability

Data produced and used in this study were deposted and are stored in an OSF project “TMEM184A, Sdc4 and VE-Cadherin” at https://osf.io/385bj/?view_only=0e13145f6e074f0191f36788d2ef414e.

## Supplementary Materials

The following supporting information can be downloaded at: https://www.mdpi.com/article/10.3390/cells14110833/s1, Figure S1: TMEM184A CTD and lentiviral verification in BAOECs; Figure S2: TMEM184A-Sdc4 interactions in BAOECs are abrogated with increased detergent and incubation times; Figure S3: VE-cad goat antibody verification with minimal decreases in VE-cad goat fluorescence in confluent siTMEM cells.
