# Supplementary material for: Transmembrane Protein-184A Interacts with Syndecan-4 and Rab GTPases and Is Required to Maintain VE-Cadherin Levels
Source: Cells. 2025 Jun 3;14(11):833. doi: 10.3390/cells14110833 (PMC12154307; doi:10.3390/cells14110833)
Supplement: Supplementary file 1 [file cells-14-00833-s001.zip › cells-3408376-supplementary.pdf]

Supplemental figures and legends. Cells3408376, Altenburg et al.

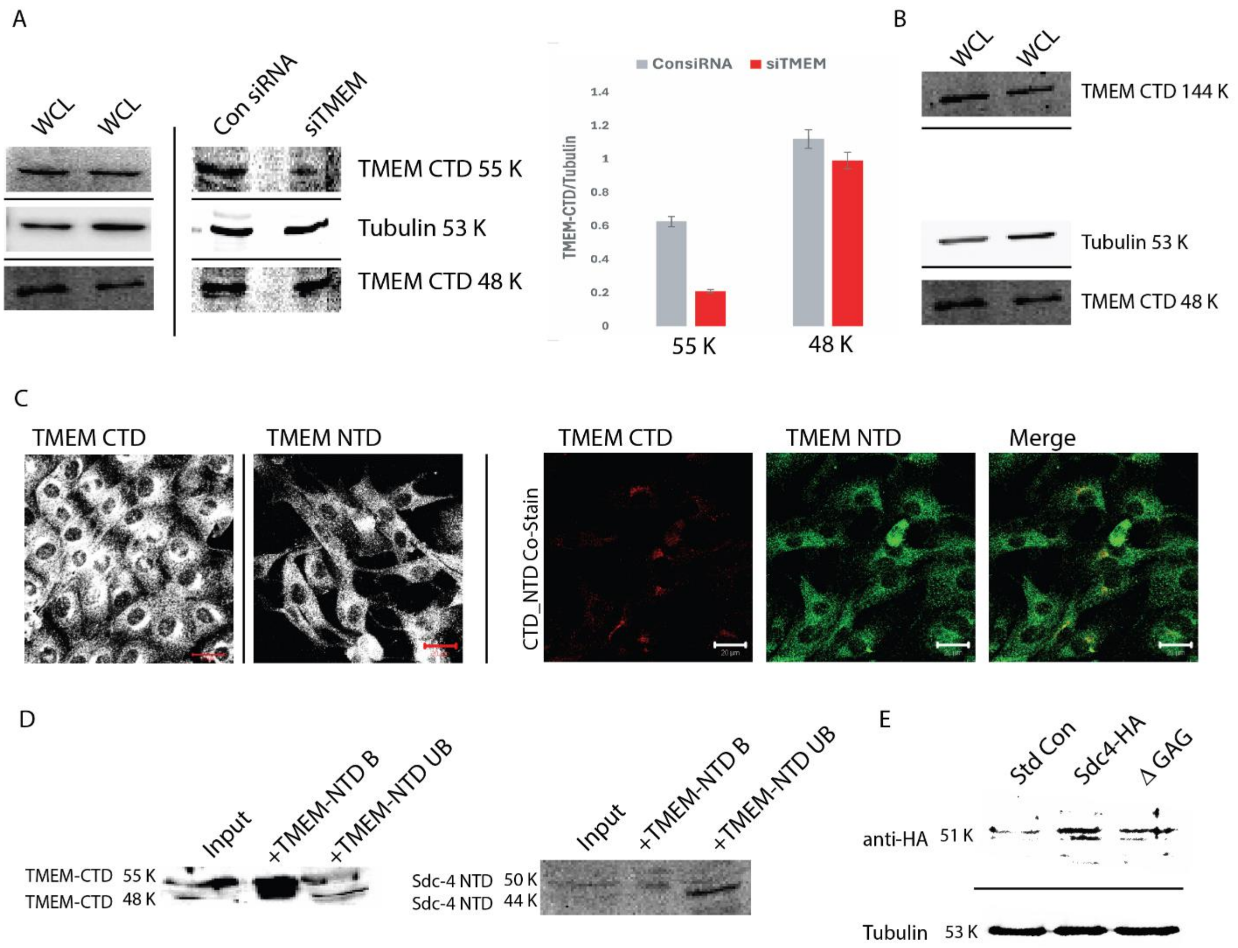

Figure S1. TMEM184A CTD and lentiviral verification in BAOECs. A. Whole Cell Lysate (WCL) TMEM184A CTD rabbit polyclonal (1:500) antibody confirmation staining (55 kDa and 48 kDa) compared to Tubulin loading control (53 kDa). In siTMEM cells, band densities normalized to Tubulin are decreased. B. A higher molecular weight band (144kDa) representing TMEM184A is also confirmed in a separate WCL harvest with a lower molecular weight band (48 kDa) compared to Tubulin loading control (53 kDa). C. IF staining of TMEM184A CTD and NTD antibodies, stained separately (left) at 1:100 dilutions and co-stained (right) at 1:100 dilutions. D. TMEM184A NTD pull down confirmation with TMEM184A CTD polyclonal staining shown in A (left). The same blot stained with the Sdc4 mouse monoclonal (right) shows the reciprocal of the pull down in Figure S1B. E. Lentiviral construct verification in Sdc4-HA and Sdc4-HA-ΔGAG lines compared to standard control cells using anti-HA goat polyclonal (1:1000) antibody (51 kDa) compared to Tubulin loading control (53 kDa).

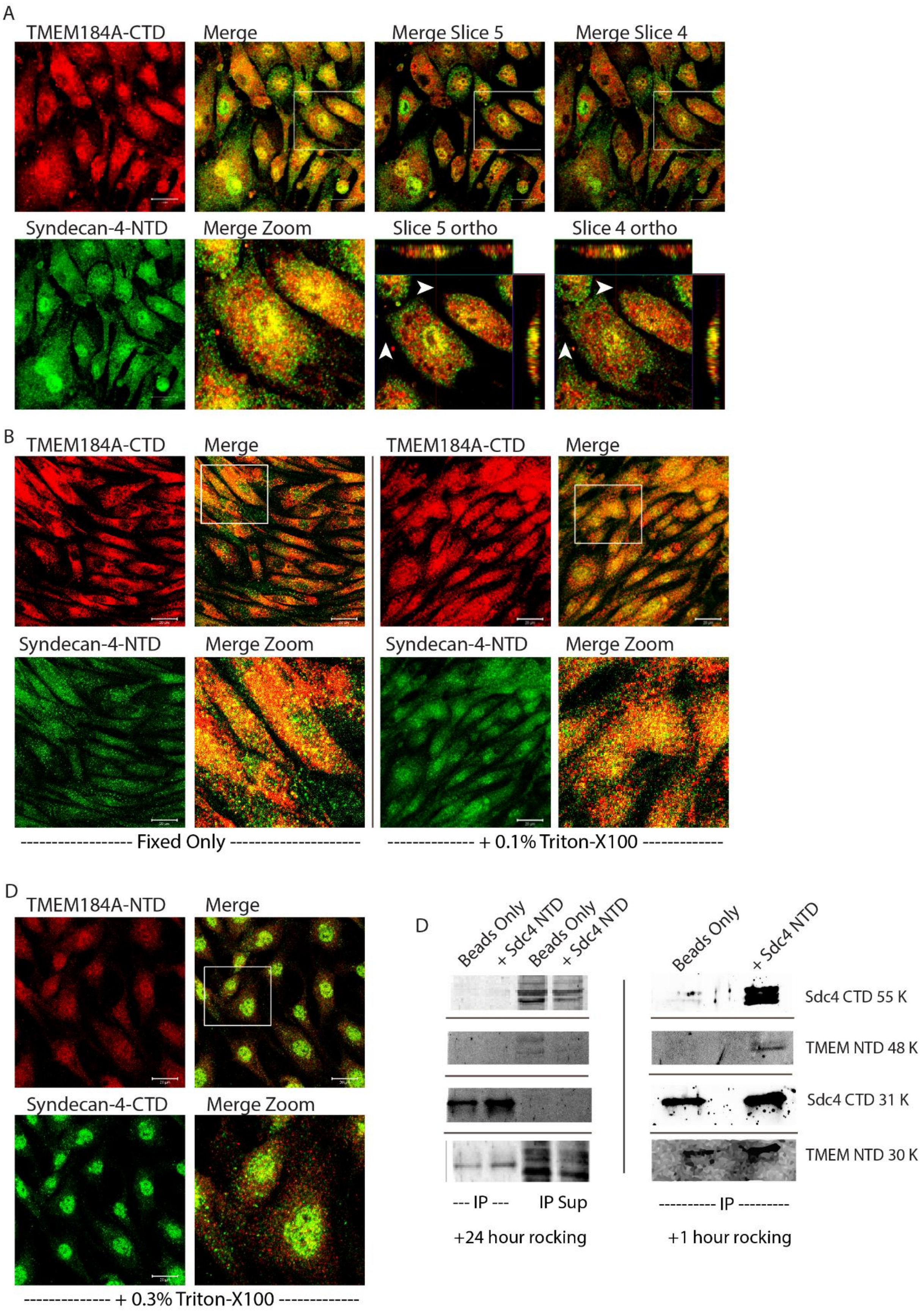
A
TMEM184A-CTD
Merge
Merge Slice 5
Merge Slice 4
Syndecan-4-NTD
Merge Zoom
Slice 5 ortho
Slice 4 ortho
B
TMEM184A-CTD
Merge
TMEM184A-CTD
Merge
Syndecan-4-NTD
Merge Zoom
Syndecan-4-NTD
Merge Zoom
------------------ Fixed Only --------------------
------------- + 0.1% Triton-X100 -------------
D
TMEM184A-NTD
Merge
Syndecan-4-CTD
Merge Zoom
-------------- + 0.3% Triton-X100 -------------
D
Beads Only
+ Sdc4 NTD
Beads Only
+ Sdc4 NTD
Beads Only
+ Sdc4 NTD
Sdc4 CTD 55 K
TMEM NTD 48 K
Sdc4 CTD 31 K
TMEM NTD 30 K
--- IP ---
IP Sup
---------- IP ---------
+24 hour rocking
+1 hour rocking

Figure S2. TMEM184A-Sdc4 interactions in BAOECs are abrogated with increased detergent and incubation times. A. Reciprical IF staining of TMEM184A CTD (red) merged with Sdc4 NTD (green) in 0.1% Triton X-100 permeabilized conditions with merged maximum intensity (Max) and orthogonal (ortho) projections of z-slices 5 and 4 and Max and ortho zoom (white boxes) views (white arrowheads denote x and y coordinates of z-slices). Scale 20 µm. B. IF staining of TMEM184A CTD (red) and Sdc4 NTD (green) in fixed only and 0.1% Triton X-100 permeabilized conditions. Scale 20 µm. Ten images across two independent experiments from each condition were compared. C. IF staining comparison of TMEM184A NTD (red) and Sdc4 CTD (green) in 0.3% Triton X-100 permeabilized conditions. Scale 20 µm. D. Comparison of bound and unbound fractions of 24 hour and 1 hour +Sdc4 NTD mouse monoclonal antibody incubations in western blotting in BAOEC cell lysate stained with anti-Sdc4 CTD rabbit (56 and 44 kDa doublet) and TMEM184A NTD (46 kDa band). Additional red gel bead shed is shown at 35 kDa with Sdc4 monomer (31 K) and TMEM NTD cleavage products (30 K). Sdc4 IP and WB was obtained in duplicate.

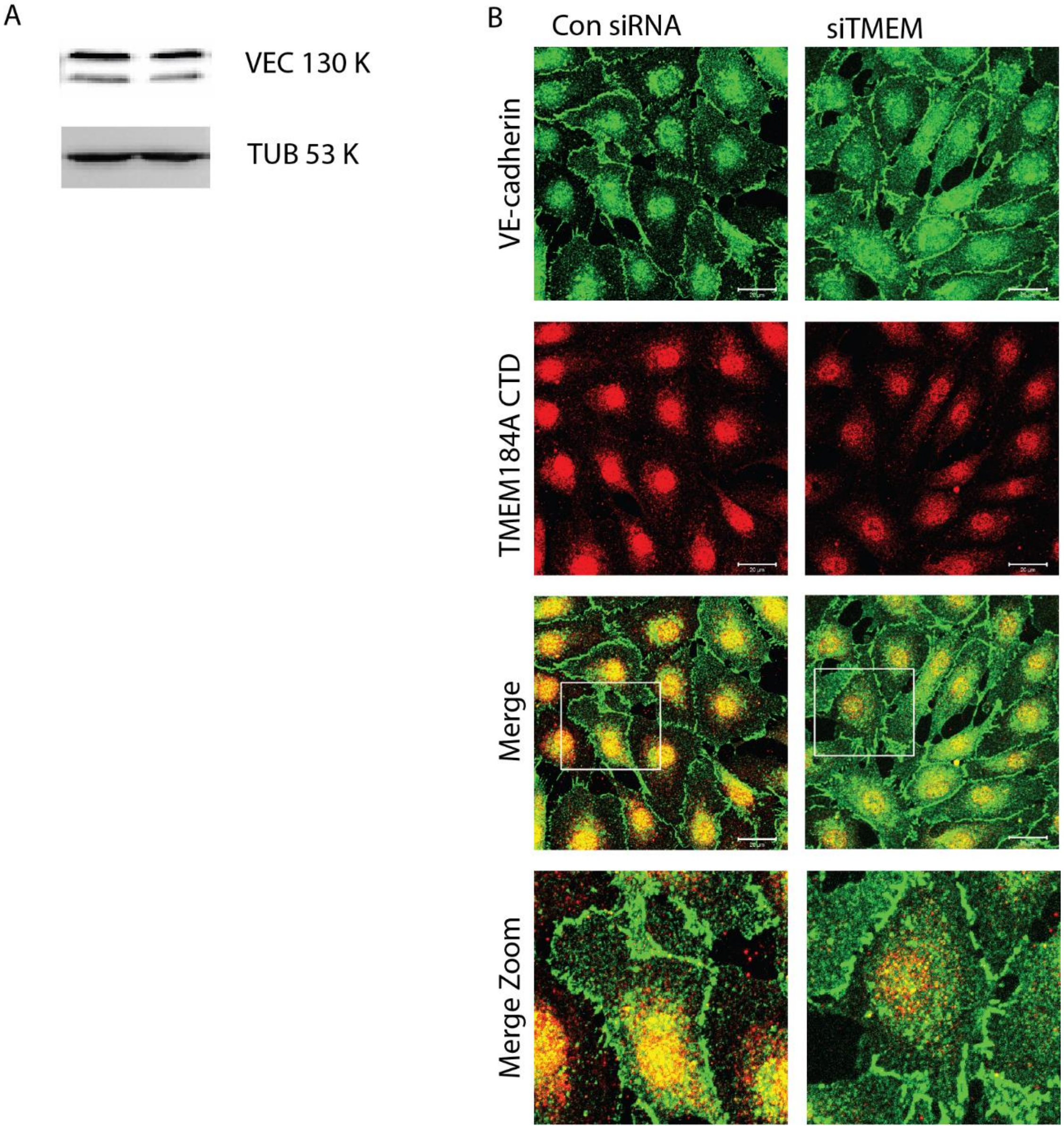

Figure S3. VE-cad goat antibody verification with minimal decreases in VE-cad goat fluorescence in confluent siTMEM cells. A. WB of WCL stained with VE-cad goat polyclonal (130 kDa), Tubulin (53 kDa). B. Representative images of IF staining of VE-cad (green) and TMEM184A CTD (red) in confluent control and siTMEM cell groups quantified in Figure 3B.
